# Exposure of Dentists to *Mycobacterium tuberculosis*, Ibadan, Nigeria

**DOI:** 10.3201/eid1609.100447

**Published:** 2010-09

**Authors:** Simeon I. Cadmus, Victoria N. Okoje, Babafemi O. Taiwo, Dick van Soolingen

**Affiliations:** Author affiliations: University of Ibadan, Ibadan, Nigeria (S.I. Cadmus, V.N. Okoje);; Northwestern University Feinberg School of Medicine, Chicago, Illinois, USA (B.O. Taiwo);; Radboud University Nijmegen, Nijmegen, the Netherlands (D. van Soolingen);; National Institute for Public Health and the Environment, Bilthoven, the Netherlands (D. van Soolingen)

**Keywords:** Running Head: Dentists and *M. tuberculosis*, Nigeria, Mycobacterium tuberculosis, dental facility, dental patients, public health, risk, bacteria, exposure, Nigeria, tuberculosis and other mycobacteria, dispatch, *Suggested citation for this article:* Cadmus SI, Okoje VN, Taiwo BO, van Soolingen D. Exposure of dentists to *Mycobacterium tuberculosis*, Ibadan, Nigeria. Emerg Infect Dis [serial on the Internet]. 2010 Sep [*date cited*]. http://dx.doi.org/10.3201/eid1609.100447

## Abstract

To determine the prevalence of *Mycobacterium* *tuberculosis* infection among dental patients and to assess dentists’ risk for exposure, we conducted a study among dental patients at a large tertiary hospital in Nigeria, a country where tuberculosis is endemic. Ten (13%) of 78 sputum samples obtained were positive for *M. tuberculosis*.

Tuberculosis (TB) is a serious public health concern globally, and almost half of new infections are undetected (*1*). The infection is spread by airborne droplet nuclei that contain *Mycobacterium tuberculosis*, which remain airborne for minutes to hours after expectoration through coughing, sneezing, or talking by persons with pulmonary TB (*2*).

Acquisition of TB in healthcare facilities is a well-recognized hazard for healthcare workers and patients. Dental practitioners may be at increased risk because they work in close proximity to potentially infectious secretions (*3*). The risk is higher in areas like Nigeria, where TB is endemic; control practices are poor; and compliance with guidelines for preventing TB in healthcare facilities, such as those issued by the US Centers for Disease Control and Prevention (*4*), is limited.

We initiated this study to assess the risk for exposure of dentists to *M. tuberculosis* infection and to determine its prevalence among dental patients. Participants were patients in the dental clinic of University College Hospital, Ibadan, Nigeria, the largest hospital in the country.

## The Study

This study was conducted in the dental center of University College Hospital, a tertiary hospital located in Ibadan, Oyo State, southwestern Nigeria. Nigeria, which has a population of >140 million, is rated as fourth among TB-endemic nations, with a prevalence of 521 cases per 100,000 population during 2007 (*5*). Ethical clearance for the study was obtained from the Oyo State Ethical Review Board as part of a multifocused study of TB in the state.

The dental center comprises 7 units that provide care to ≈1,800 patients annually. This study was carried out in the Oral Surgery Clinic, one of the units in the center where a member of the study team is a consultant. Patients were recruited during February 2006 through July 2007. A total of 312 consecutive patients who came to the clinic for treatment were invited to participate; 101 gave consent (23/101 patients were excluded because they could not produce a sputum sample). Thus, 78 patients were evaulated.

Each participant completed a structured questionnaire that requested information about age, sex, occupation, bacille Calmette-Guérin (BCG) vaccination, history of chronic cough or TB, HIV status, and contact with a patient who had chronic cough or TB. A single expectorated sputum sample was collected from each participant and placed in a sterile universal container. Collection was done through the support of medical house officers/registrars posted to the unit; a consultant (1 of the study group members) was responsible for overall supervision.

The sputum specimen and culture were processed by using a standard procedure as described by Cadmus et al. (*6*). Using the Ziehl-Neelsen technique, we performed smear microscopy. Isolates were harvested for molecular typing analysis by scraping the growth from a slope into 200 µL of distilled water and heating the product at 80ºC for 1 h.

Molecular identification of isolates was conducted in the Veterinary Laboratories Agency, Addlestone, Surrey, UK, by spoligotyping as described by Kamerbeek et al. (*7*) with minor modifications and repeated at the Center for Infectious Disease Control (RIVM) in the Netherlands. Furthermore, the Hain Genotype *Mycobacterium tuberculosis* complex (MTBC) test (Hain Lifescience, Nehren, Germany) was applied to determine whether an isolate was a member of the MTBC, after which the Hain line probe assay identification kit for subspecies of MTBC was applied (Hain Lifescience).

Seventy-eight dental patients who could give a sputum sample consented to participate in the study. Forty-one (52%) participants were women; median age was 32 years. A total of 20 participants (26%) reported having received BCG vaccine, and 10 reported prior exposure to a person with chronic cough or with TB. None of the participants reported a personal history of chronic cough or TB. Ten (13%) of the 78 participants had culture-confirmed TB, 7 of whom had initial positive acid-fast bacilli (AFB) smears through concentrated sputum samples. The HIV status of 70 respondents (including those with TB) was unavailable, and 1 of the remaining 8 respondents reported being positive for HIV during questionnaire/sample collection. The demographic characteristics, AFB smear results, BCG vaccination history, and known TB exposure of the participants with mycobacterial growth are presented in the Table.

**Table:** Demographic characteristics, BCG vaccination status, and TB exposure history of participants with positive sputum culture results, University College Hospital, Ibadan, Nigeria, 2006–2007*

Participant ID	Age, y/sex	Occupation	BCG vaccination	Exposure to TB patients	Positive AFB smear
1	49/F	Hairdresser	No	No	Yes
2	60/M	Trader	Yes	No	Yes
3	24/F	None	Yes	No	No
4	30/F	Student	No	No	Yes
5	45/M	Businessman	Yes	No	Yes
6	77/M	Retired	Yes	No	No
7	44/M	Clergyman	Yes	No	No
8	40/F	Trader	No	Yes	Yes
9	63/M	Retired	Yes	No	Yes
10	54/F	Civil servant	Yes	No	Yes

*BCG, bacille Calmette-Guérin; TB, tuberculosis; ID, identification; AFB, acid-fast bacilli.

Spoligotyping in the United Kingdom and repeated molecular identification in the Netherlands identified the strains as *M. tuberculosis*. In spoligotyping, different genotypic patterns were visualized (*8*), which limited the possibility of laboratory cross-contamination.

## Conclusions

In patients who sought treatment at the dental clinic of a tertiary hospital in Nigeria, we found an unexpectedly high rate of unrecognized infection with pathogenic mycobacteria. Ten (13%) of 78 study participants who provided sputum samples were infected with *M. tuberculosis*. Our findings corroborate other studies of TB cases and show that AFB smears alone would miss some infected patients. *M. tuberculosis* is transmitted with high efficiency to household contacts (*9*), and transmission of mycobacteria, including multidrug-resistant *M. tuberculosis* strains, from dental patients to dental practitioners, probably occurs (*10*). Accordingly, infected participants in this study, especially those who had positive AFB smears, were, in principle, capable of infecting dental practitioners and other patients. Reports from other settings suggest that the threat of TB transmission from clients to dentists is not only theoretical (*11*). This extremely high percentage of undiagnosed AFB positivity is of public health concern because dental clinics in TB-endemic areas are not usually considered a place of high risk for TB transmission; therefore, preventive measures are not routinely implemented.

This study has several limitations. First, patients were recruited from only 1 of the 7 units in the dental center, constricting the pool of patients invited to participate. However, all patients visiting the center were exposed to similar conditions, and the patients who came to the oral surgery clinic had often been referred from other units in the dental center. Second, only 1 sputum sample was collected from each participant. The yield possibly would have been higher if we had collected more samples. Third, dental problems (*12*) and TB (*13*) are major comorbidities among persons infected with the HIV, and the synergism between *M. tuberculosis* and HIV is well documented**.** Because we did not collect complete histories on the HIV status of participants, we are unable to delineate the role of HIV with respect to our observations. However, in 2003, the same clinic recorded a 2.3% HIV prevalence among 300 patients screened for oral surgery (*14*); the 2008 prevalence data for the state was 2.2% (*15*). Fourth, our study was conducted in the dental clinic of the major tertiary hospital in 1 of the largest cities in Nigeria; thus, the findings may not be generalizable to all facilities where dental care is provided. Nevertheless, a surprisingly high proportion of dental patients in this study had potentially infectious TB. Therefore, evidence-based strategies should be implemented to quickly diagnose TB and prevent its transmission in dental clinics. Of particular importance in preventing nosocomial TB in dental clinics will be the adoption of infection control measures that may include the use of appropriate masks. Finally, our study also emphasizes the view that active case finding in high prevalence settings could be beneficial.
